# AMBITION-*cm*: intermittent high dose AmBisome on a high dose fluconazole backbone for cryptococcal meningitis induction therapy in sub-Saharan Africa: study protocol for a randomized controlled trial

**DOI:** 10.1186/s13063-015-0799-6

**Published:** 2015-06-17

**Authors:** Mooketsi Molefi, Awilly A. Chofle, Síle F. Molloy, Samuel Kalluvya, John M. Changalucha, Francesca Cainelli, Tshepo Leeme, Nametso Lekwape, Drew W. Goldberg, Miriam Haverkamp, Gregory P. Bisson, John R. Perfect, Emili Letang, Lukas Fenner, Graeme Meintjes, Rosie Burton, Tariro Makadzange, Chiratidzo E. Ndhlovu, William Hope, Thomas S. Harrison, Joseph N. Jarvis

**Affiliations:** Department of Family Medicine and Public Health, Faculty of Medicine, University of Botswana, P.O.Box 1357 ABG, Gaborone, Botswana; Botswana-Upenn Partnership, Gaborone, Botswana; Bugando Medical Centre, Mwanza, Tanzania; National Institute for Medical Research, Mwanza Research Centre, Mwanza, Tanzania; Research Centre for Infection and Immunity, St. George’s University of London, London, UK; Division of Infectious Diseases, Department of Medicine, Perelman School of Medicine, University of Pennsylvania, Philadelphia, USA; Division of Infectious Diseases, Department of Medicine, Duke University Medical Center, Durham, NC USA; Swiss Tropical and Public Health Institute, Basel, Switzerland; Ifakara Health Institute, Ifakara, Morogoro Tanzania; ISGLOBAL, Barcelona Ctr. Int. Health Res. (CRESIB), Hospital Clinic-Universitat de Barcelona, Barcelona, Spain; Institute of Infectious Disease and Molecular Medicine and Department of Medicine, University of Cape Town, Cape Town, South Africa; Department of Medicine, Imperial College London, London, UK; Department of Medicine, Khayelitsha Hospital, Khayelitsha, Cape Town, South Africa; Ragon Institute of MGH, MIT, Harvard Cambridge, MA USA; Department of Medicine, University of Zimbabwe College of Health Sciences, Parirenyatwa Hospital, Harare, Zimbabwe; Department of Molecular and Clinical Pharmacology, Institute of Translational Medicine, University of Liverpool, Liverpool, UK; Department of Clinical Research, Faculty of Infectious and Tropical Diseases, London School of Hygiene and Tropical Medicine, London, UK

**Keywords:** Cryptococcal meningitis, HIV, AmBisome, Amphotericin B, Fluconazole, Clinical trial

## Abstract

**Background:**

Cryptococcal meningitis (CM) is a leading cause of mortality among HIV-infected individuals in Africa. Poor outcomes from conventional antifungal therapies, unavailability of flucytosine, and difficulties administering 14 days of amphotericin B are key drivers of this mortality. Novel treatment regimes are needed. This study examines whether short-course high-dose liposomal amphotericin B (AmBisome), given with high dose fluconazole, is non-inferior (in terms of microbiological and clinical endpoints) to standard-dose 14-day courses of AmBisome plus high dose fluconazole for treatment of HIV-associated CM.

**Methodology/design:**

This is an adaptive open-label phase II/III randomised non-inferiority trial comparing alternative short course AmBisome regimens. Step 1 (phase II) will compare four treatment arms in 160 adult patients (≥18 years old) with a first episode of HIV-associated CM, using early fungicidal activity (EFA) as the primary outcome: 1) AmBisome 10 mg/kg day one (single dose); 2) AmBisome 10 mg/kg day one and AmBisome 5 mg/kg day three (two doses); 3) AmBisome 10 mg/kg day one, and AmBisome 5 mg/kg days three and seven (three doses); and 4) AmBisome 3 mg/kg/d for 14 days (control); all given with fluconazole 1200 mg daily for 14 days. STEP 2 (phase III) will enrol 300 participants and compare two treatment arms using all-cause mortality within 70 days as the primary outcome: 1) the shortest course AmBisome regimen found to be non-inferior in terms of EFA to the 14-day control arm in STEP 1, and 2) AmBisome 3 mg/kg/d for 14 days (control), both given with fluconazole 1200 mg daily for 14 days. STEP 2 analysis will include all patients from STEP 1 and STEP 2 taking the STEP 2 regimens. All patients will be followed for ten weeks, and mortality and safety data recorded. All patients will receive consolidation therapy with fluconazole 400–800 mg daily and ART in accordance with local guidelines. The primary analysis (for both STEP 1 and STEP 2) will be intention-to-treat.

**Trial registration:**

ISRCTN10248064. Date of Registration: 22 January 2014

## Background

Cryptococcal meningitis is a major cause of death in Sub-Saharan Africa, causing 10–20 % of all deaths in HIV infected cohorts, resulting in up to 500,000 deaths annually [1]. The poor outcomes reported using currently available antifungal therapy in African centres are a critical driver of this high mortality [2–6], and provide the impetus to develop novel treatment regimens for cryptococcal meningitis. Prior studies demonstrate the need for rapid initial clearance of cryptococcal infection for survival [2]. Conventional amphotericin B deoxycholate (AmB-d) induction for 2 weeks, given with oral flucytosine (5FC), is rapidly effective and still the gold standard [7–10], but safe delivery is not feasible or sustainable in most centres in Africa due to the need for 2 weeks hospitalisation and intravenous (iv) access, the cost of extra iv fluids and electrolytes, and above all, the availability and costs of rapid, reliable laboratory monitoring. Phlebitis with secondary infection [11], renal impairment, and anaemia are also significant problems with 14 days induction therapy with conventional AmB-d. Flucytosine is prohibitively expensive and not available in Africa [12, 13]. The alternative treatment, oral fluconazole, even at a dosage up to 1200 mg/d, as used currently in many centres in Africa, is still much less rapidly fungicidal than AmB-d, and mortality at 10 weeks remains in excess of 50 % [5, 14, 15].

**Fig. 1:**
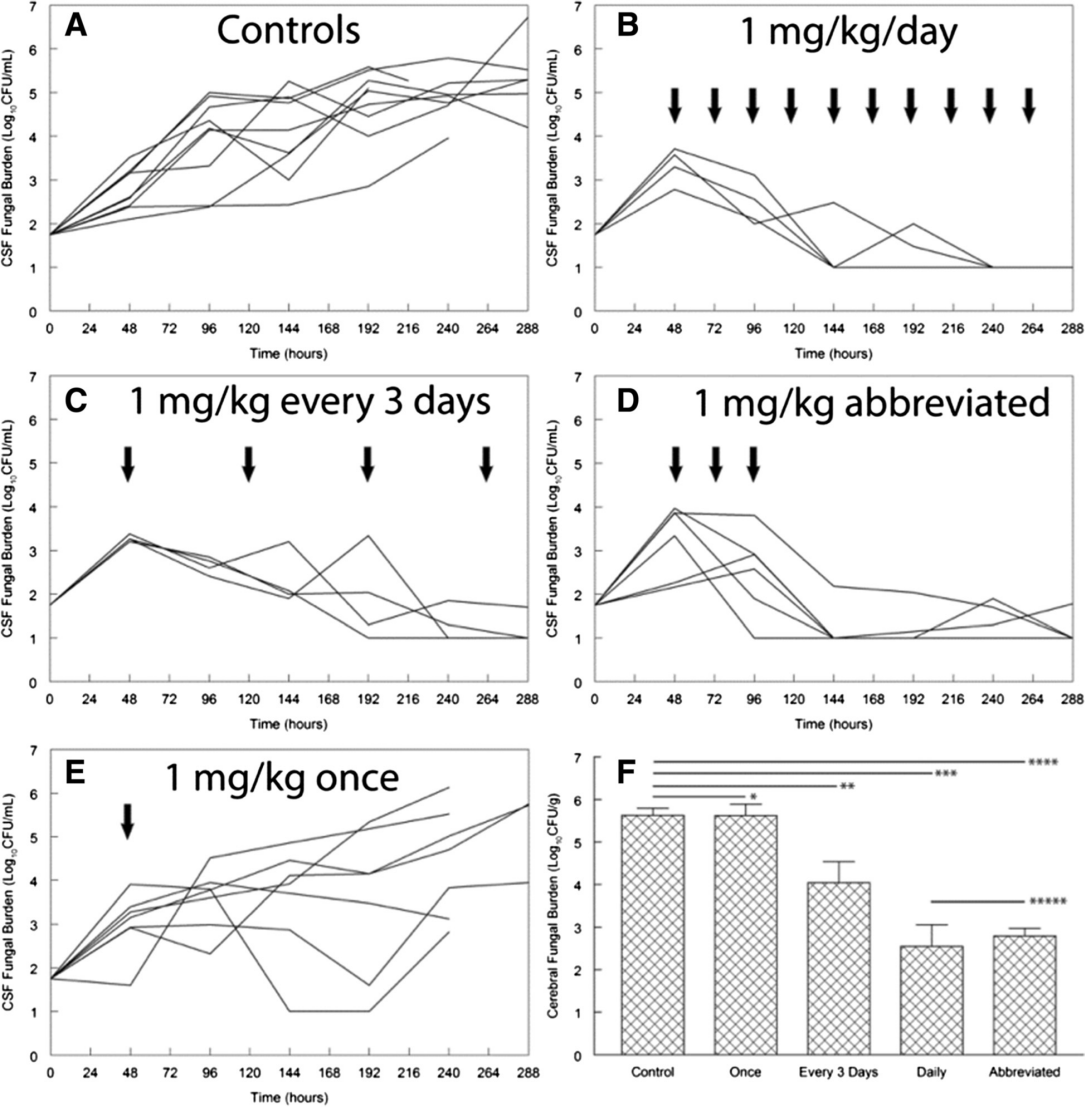
The effect of various regimens of amphotericin B for experimental cryptococcal meningoencephalitis. Amphotericin B deoxycholate 1 mg/kg was administered daily (**b**), every third day (**c**), as an abbreviated regimen for 3 consecutive days (**d**) and once (**e**). The solid arrows show the time of drug administration. The data in **a**-**e** represent the time course of infection in the cerebrospinal fluid (*CSF*). Each line represents the fungal burden in an individual rabbit. **f** shows the fungal density in the cerebrum at the end of the experiment. Each bar represents the mean fungal density ± standard error of the mean. There is no statistically significant difference in the cerebral fungal density of rabbits receiving daily therapy versus those receiving an abbreviated regimen of 1 mg/kg at 48, 72 and 96 h relative to inoculation: *p = 1.00 (not significant), **p = 0.006, ***p = <0.001, ****p = <0.001, *****p = 1.00 (not significant). *CFU*, colony-forming units. From Livermore J, Howard SJ, Sharp AD, et al. Efficacy of an abbreviated induction regimen of amphotericin B deoxycholate for cryptococcal meningoencephalitis: 3 days of therapy is equivalent to 14 days. MBio 2013; 5 (1): e00725-13. With permission

In recent studies, using shorter-course conventional AmB-d (5–7 days) with high dose fluconazole [16, 17], no slowing of the rate of clearance of infection over the first 14 days was seen compared with 14 days of AmB-d, and there was a large gain in tolerability and therefore, ability to implement. The sustained effect of short-course AmB-d may be related to its prolonged mean residence time in brain tissue [18], and animal models suggest that abbreviated regimens of AmB-d (as short as 3 days) are as effective as full 14-day courses (Fig. 1) [18]. These data support the concept of short but still highly effective induction regimens.

However, liposomal amphotericin B (AmBisome) would be even more suitable for this strategy than conventional AmB-d: it is less nephrotoxic, allowing much higher doses to be given safely, and also has excellent tissue penetration [19] and a long tissue half-life [20–23], raising the possibility of delivering highly effective induction therapy with very few (one to three) doses. Single doses of up to 15 mg/kg have been safely given to patients [22], and doses of 10 mg/kg are routinely given and have been shown to be efficacious for treatment of visceral leishmaniasis [24] and other invasive fungal infections [25].

Although AmBisome is recommended as a first-line agent for treating cryptococcal meningitis in several national guidelines [9, 26], there is uncertainty about the optimal dosing strategies [27]. Pharmacokinetic data from animal models [21] and humans [20] suggest that increasing AmBisome dose from the currently recommended 3–4 mg/kg may be required to facilitate safe, easy to administer, and effective intermittent dosing regimens and produce an antifungal effect that is as least as good as standard daily therapy [21]. High-dose fluconazole could be started with AmBisome, given evidence from randomised studies of an additive effect of high-dose fluconazole with conventional AmB-d [8, 28, 29], and to provide ongoing consolidation therapy.

**Fig. 2:**
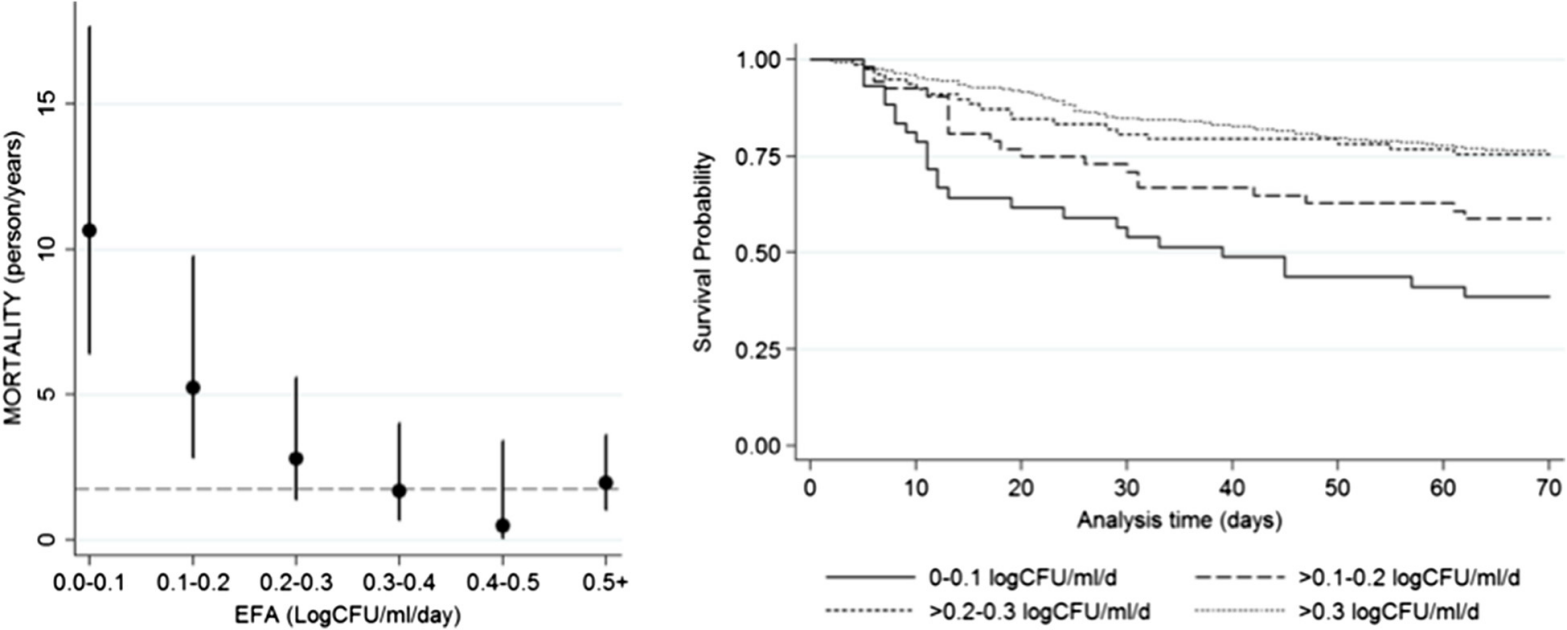
Early fungicidal activity and mortality. The left hand panel shows the crude mortality rate (per person year) calculated over the first 2 weeks of treatment according to early fungicidal activity (EFA) in the 450 patients of the 501 anti-retroviral therapy (ART)-naive patients recruited in the studies listed in Reference 2 with slope (EFA) data. Patients have been stratified by EFA, with the slowest rate of clearance being 0–0.1 log_10_CFU/ml/day (equating to a 0 to 0.1 log drop in colony-forming units (*CFU*) per ml of cerebrospinal fluid (*CSF*) per day) and the highest rate of clearance being 0.5+ (equating to a half-log_10_ drop or more in CFU counts/ml of CSF per day). The right hand panel shows Kaplan Meier survival curves for the 501 ART-naive patients recruited into the studies listed in Reference 2 over the first 10 weeks of treatment, stratified by EFA. The slowest rates of clearance are indicated by the *solid black line* (EFA 0–0.1 decline in log_10_CFU/ml/day) and the most rapid rates of clearance are indicated by the small *dotted line* (EFA >0.3 decline in log_10_CFU/ml/day)

Prior to any expanded access programme for AmBisome for cryptococcal infection, it is vital that we define the most clinically effective and most cost-effective schedules for its use. This study will provide the data needed. We propose to initially use early fungicidal activity (EFA), a highly efficient method to reliably test alternative treatment schedules in relatively small numbers of patients [2, 7, 14, 16, 17, 28, 30–34], to determine the fungicidal activity of several short-course dosing regimens. In addition to being a statistically powerful endpoint, EFA had been consistently shown to be independently associated with clinical outcome (Fig. 2) [2, 34]. After EFA evaluation in 160 patients, EFA, safety data and clinical efficacy data will be used to select the best performing intermittent dosing arm for ongoing clinical endpoint comparison with standard daily dosing. The principal aim of this study is to examine whether short-course high-dose AmBisome treatment, given with high-dose fluconazole, is non-inferior in terms of both microbiological and clinical endpoints to standard-dose 14-day courses of AmBisome plus high-dose fluconazole.

## Method/design

### Study design

This is an adaptive open label phase II/III randomised non-inferiority trial to compare alternative short course AmBisome regimens for the treatment of HIV-associated cryptococcal meningitis.

### Main hypothesis

Short-course high-dose AmBisome given with high dose fluconazole will be non-inferior to 2 weeks of daily dosed AmBisome-based induction therapy for the treatment of HIV-associated cryptococcal meningitis.

### Objectives

In step I (phase II), the primary objective is to determine the EFA of three alternative schedules of intermittent high-dose AmBisome in comparison with standard daily AmBisome, all given with fluconazole at 1,200 mg/d for the first 2 weeks, in induction therapy for HIV-associated cryptococcal meningitis. Secondary objectives are to determine the pharmacokinetic (PK) parameters and PK/pharmacodynamic (PD) associations of alternative schedules of intermittent high-dose AmBisome, to gather data on the clinical efficacy and safety of these alternative schedules of intermittent high-dose AmBisome in induction therapy for HIV-associated cryptococcal meningitis and to model the cost-effectiveness of these alternative schedules of intermittent high-dose AmBisome in induction therapy for HIV-associated cryptococcal meningitis. In step II (phase III), which includes transitioning to a phase III comparison of the shortest course AmBisome arm from step 1 meeting predefined minimum safety and efficacy standards with standard daily AmBisome dosing, the primary objective is to determine mortality within the first 10 weeks using short course high-dose AmBisome in comparison with standard daily AmBisome (for 14 days) for induction therapy of HIV-associated cryptococcal meningitis, both given with fluconazole at 1,200 mg/d for the first 2 weeks. Secondary objectives are to determine the EFA of alternative AmBisome schedules and culture status at 2 weeks, to examine the proportions of patients in each arm suffering clinical and laboratory-defined grade III/IV adverse events; median percentage change from baseline in laboratory-defined parameters by treatment arm and to determine health service costs by treatment arm.

### Setting

Step 1 will be carried out in Princess Marina Hospital (PMH), Gaborone, Botswana, which is a 500-bed tertiary care hospital in the capital city, and Bugando Medical Centre (with Sékou Touré Hospital and Butimba Hospital), Mwanza, Tanzania, which is the referral and teaching hospital for the Lake and Western zones of Tanzania, serving a population of 13 million. Step 2 will include the above plus Ifakara Health Institute, St Francis Referral Hospital, Ifakara, Tanzania, a 370-bed principal health care facility in the Kilombero district, Morogoro region, serving a catchment area of 400,000 people; Khayelitsha Hospital, with 150 adult beds, situated in the area with the highest HIV prevalence in the Western Cape, South Africa; and the Parirenyatwa General Hospital (Harare, Zimbabwe), which is one of the largest tertiary academic teaching hospitals in Zimbabwe and is affiliated with the University of Zimbabwe College of Health Sciences. A further southern African site is still to be confirmed.

### Outcome measures

In step 1 the primary outcome measure is EFA with alternative AmBisome schedules, and the secondary outcome measures include mortality at 2 and 10 weeks and time to death by treatment arm, unadjusted and adjusted for possible confounders and PK and PK/PD parameters. The PK-PD data will be modelled using population methodology, enabling a robust estimate of drug exposure in each patient, which can then be related to the observed pharmacodynamic data (e.g., the rate of decline in fungal density, or the time to undetectable cultures). Proportions of patients in each arm suffering clinical and laboratory-defined grade III/IV adverse events, and median percentage change from baseline in laboratory-defined parameters by treatment arm, and health service costs by treatment arm, will be determined. In step 2 the primary outcome measure will be mortality within 10 weeks by treatment arm. Secondary analysis will be performed with adjustment for possible confounders. EFA with alternative AmBisome schedules and culture status at 2 weeks, the proportion of patients in each arm suffering clinical and laboratory-defined grade III/IV adverse events, median percentage change from baseline in laboratory-defined parameters, and health service costs, will be evaluated by treatment arm as secondary outcome measures.

### Sample size

Step 1: using a non-inferiority design, assuming an EFA of 0.50 log colony forming units (CFU)/d, with standard deviation of 0.25 in the standard daily dosing control arm, an acceptable delta of 0.2, one-sided alpha of 0.025 and 90 % power, gives a sample size of 33 patients per arm. A total sample of 40 patients per arm (160 patients in total) will therefore be planned to allow for patients who die prior to having a second lumbar puncture (LP) to allow for an EFA measurement. An interim analysis will be done after 20 patients per arm to make adjustments, if necessary, to the required sample size, or to consider stopping one or more arms if any are found to result in significantly lower EFA than the control arm (*p* <0.05) and are deemed unlikely to meet the predefined non-inferiority criteria outlined above if continued to completion.

Step 2: a non-inferiority design has been chosen for assessment of the primary mortality endpoint, as a short course of AmBisome is not expected to be superior to the standard course, but is expected to have similar efficacy, while being easier to administer, affordable, and better tolerated. Using a non-inferiority design assuming 10-week mortality of 25 %, with an acceptable non-inferiority margin of 15 %, one-sided α = 0.025 and 90 % power, gives a sample size of 176 per arm. Using the 40 patients already recruited into each of the control arm and the short-course AmBisome arm from step 1 chosen for step 2, 136 additional patients per arm are required. To allow for withdrawals and losses to follow up a sample size of 150 patients per arm is planned, giving a total sample size for step 2 of 300 patients.

### Choice of non-inferiority margin

Step 1: previous studies demonstrate that the EFA of 14-day high-dose amphotericin-based regimens is in the region of 0.5 log CFU/d [31], and it is anticipated that standard-dose AmBisome regimens will be similar. The acceptable delta of 0.2 log CFU/d was selected on the basis of combined EFA data from over 500 patients included in prior studies (Fig. 2) suggesting that notable increases in mortality are not seen until the EFA drops below 0.3 log CFU/d.

Step 2: we anticipate that short-course AmBisome will have the same 10-week mortality as standard 14-day courses. A non-inferiority margin of 15 % below an assumed efficacy of 75 % (based upon recent data from South Africa and Botswana [3, 28, 35]) was considered acceptable based on the clinical acceptability and feasibility of the novel short-course regimens compared to standard 14-day courses of AmBisome, the current limited availability of conventional Amphotericin B-based treatments in much of Africa, reported treatment outcomes with Amphotericin B monotherapy [36] and high-dose fluconazole monotherapy in Africa [5, 14], and considerations relating to achievable recruitment rate projections. The Data Monitoring Committee (DMC) will regularly review mortality data, and appropriate increases to sample size will be made should the mortality in the control arm prove to be higher than anticipated.

### Inclusion and exclusion criteria

Patients to be included in the study are consecutive patients ≥18 years with a first episode of cryptococcal meningitis confirmed by either India ink or cryptococcal antigen test (CrAg) in the cerebrospinal fluid (CSF). They should be known to be HIV positive or willing to undertake an HIV test (which is positive) and agree to participate in the study. Pregnant (confirmed by urinary pregnancy test) or lactating patients, patients with a previous serious reaction to study drugs, or patients on antifungal treatment for more than 48 h or concomitant medication that is contraindicated with study drugs at the time of assessment for enrolment in the study, will be excluded. ART-naïve and experienced patients will be enrolled.

### Consent

Written informed consent to enter into the trial must be obtained from participants, or in the case of those with mental obtundation, from the patient’s guardian or a person with legal responsibility, after explanation of the aims, methods, benefits and potential hazards of the trial and before any trial-specific procedures are performed or any blood is taken for the trial. In the case of patients with altered mental status enrolled into the study, consent will be obtained as above as soon as the patient’s mental status improves, with care taken to ensure they understand that they are free to withdraw from the study and if they do so this will not jeopardise their immediate and future care. Participants who withdraw will revert to standard of care at the recruiting site (usually amphotericin B deoxycholate and fluconazole 800 mg daily for 2 weeks, or fluconazole 1,200 mg daily for 2 weeks). It will be made completely and unambiguously clear that the participant (or guardian) is free to refuse to participate in all or any aspect of the trial, at any time and for any reason, without incurring any penalty or affecting their treatment (or that of their guardian). Signed consent forms will be kept by the investigator and documented in the case report form (CRF) and a copy given to the participant or family.

### Allocation

Patients will be randomised individually using a computer-generated programme. Randomisation lists and sealed envelopes will be prepared by an independent statistician in advance and sent to the sites. Randomisation will be stratified by abnormal mental status and ART status on admission at each site to ensure equal numbers of severely ill patients and patients on ART in each group. An allocation ratio of 1:1 and block sizes of 8 will be used. The study monitor will audit the randomization and allocation concealment process to ensure there are no violations [37].

### Interventions

**Fig. 3:**
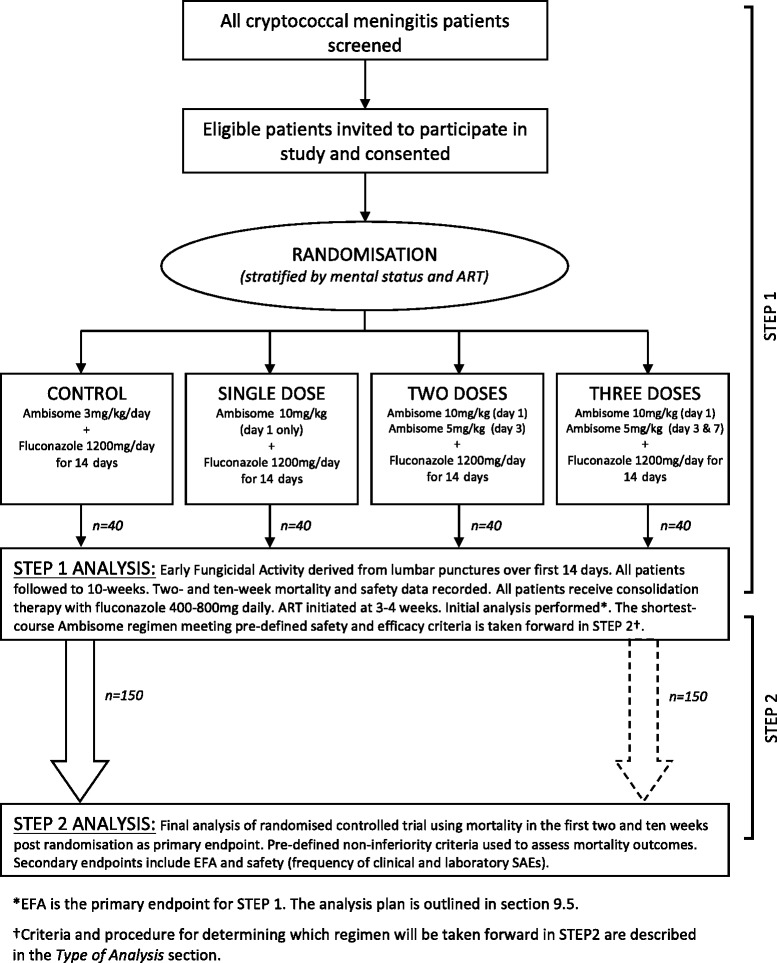
Trial schema. Trial entry, randomisation and treatment. *ART* anti-retroviral therapy, *EFA* early fungicidal activity, *SAE* serious adverse event

In step 1, the four arms of the intervention are AmBisome 10 mg/kg on day 1 (single dose), AmBisome 10 mg/kg on day 1, AmBisome 5 mg/kg on day 3 (two doses), AmBisome 10 mg/kg on day 1, AmBisome 5 mg/kg on days 3 and 7 (three doses), and AmBisome 3 mg/kg/d for 14 days, which will act as the control (Fig. 3). In step 2 where the interest is in clinical endpoints, the intervention will be use of the shortest-course AmBisome regimen tested in step 1 (either regimen 1, 2 or 3 above) meeting the predefined safety and efficacy criteria and selected by the Trial Steering Committee (TSC) for further study, and AmBisome 3 mg/kg/d for 14 days used as the control. Note that all patients also receive fluconazole 1,200 mg/d for the first 2 weeks, followed by 800 mg/d until 10 weeks, and 200 mg/d thereafter. ART will be commenced 4 to 5 weeks after initiation of antifungal therapy. The trial clinical team is not blinded to treatment allocation, however, laboratory personnel performing CFU counts are blinded to treatment allocation.

### Rescue medication

All patients will receive fluconazole from day 1 at 1200 mg/d for the first 2 weeks, making it very unlikely that CSF CFU counts will rise after the day 3 LP [5, 6]. However, in view of the single-dose AmBisome arm and to ensure that all patients receive effective induction therapy, if CFU counts go up (by any amount) after day 3, i.e., on the day-7 or day-14 LP, then the patient will switch to daily AmBisome (3 mg/kg/d) for 7 to 14 days and until the CSF is sterile. Thus, all patients will be ensured effective induction therapy.

### Schedule

Clinical response will be monitored daily for the first 2 weeks or until discharge (whichever is later), then on follow up at 3, 4, 6 and 10 weeks after the start of therapy. Every effort will be made (e.g., with mobile telephone calls and financial help with travelling expenses) to obtain accurate and complete follow-up data for 10 weeks after the start of treatment. Particular attention will be paid to ascertaining immune reconstitution reactions in ART-naïve patients after starting ART.

Five millilitres of blood will be collected on days 3, 5, 7, 10, 12, and 14 of the study for creatinine and electrolytes. Full blood count (FBC) and alanine transaminases (ALT) will be obtained on days 7 and 14 of the study. On the day of the visit to the study clinic (week 4), blood will be drawn for creatinine, electrolytes, FBC and ALT. An extra 2.5 ml of blood will be taken for PK studies at the time of monitoring blood tests, on days 1 and 3 (excluding the single-dose arm) at the end of AmBisome infusion, and then 3, 6, 8 and 24 h post infusion. Follow-up CSF examinations will be performed on days 3, 7, 14 and 21 to determine opening pressure, quantitative fungal culture (QCC), CSF for drug levels, and immune parameters. In a variation on prior EFA studies (LPs on day 1, 7 and 14), in step 1 LP will be done at day 21 and for day-14 and day-21 quantitative cultures, an extra plate will be spread with 1 ml of undiluted CSF – this will increase sensitivity tenfold, and may help differentiate the duration of the effect of AmBisome on clearance with alternative regimens. Cryptococcal clearance rates will be calculated using a summary statistic for each patient, the rate of decrease in log CFU per ml CSF per day derived from the slope of the linear regression of log CFU against time for each patient. A linear regression model will be used to compare mean rates of decline or EFA for each experimental treatment, giving summary differences with 95 % CI and significance levels. We will adjust analyses for potential confounding factors, including baseline fungal load. Note that follow-up CSF examinations for study purposes in step 2 will be performed on days 7 and 14 only.

### Type of analysis

After data cleaning, analysis will proceed according to the predesigned analysis plan. The primary analysis (for both step 1 and step 2) will be intention-to-treat analysis. Note, in step 1, patients who die prior to day 3 will not have EFA calculated and thus, will be censored from EFA analysis (modified intention-to-treat analysis – prior experience of over 500 patients studied using this method suggests EFA data will be available in at least 90 % of patients). Linear regression models will be calculated for step 1. Mean rate of decrease in CSF cryptococcal CFU (EFA) will be the dependent variable and treatment group (using the standard treatment arm as a comparator) will be the primary independent variable. The comparator groups will be compared to the standard treatment arm for non-inferiority using standard criteria with an acceptable delta of 0.2 log_10_ CFU/ml/day.

Secondary analysis will include per-protocol analysis. Following a crude analysis, adjusted analysis will be performed including covariates that may influence outcome. As per the crude analysis, comparator groups will be compared to the standard treatment arm for non-inferiority using standard criteria with an acceptable delta of 0.2 log_10_ CFU/ml/day. Adjustment will be made for baseline fungal burden, which has been found to be associated with rate of clearance in previous studies, and for CD4 cell count, giving summary differences with 95 % CI.

The decision about which regimen will be taken forward from step 1 to step 2 will be taken by the TSC in conjunction with the DMC, and will be based upon predefined safety and minimal efficacy criteria laid down in the full statistical analysis plan. Briefly the shortest-course AmBisome regimen of: 1) AmBisome 10 mg/kg on day 1 (single dose), 2) AmBisome 10 mg/kg on day 1, AmBisome 5 mg/kg on day 3 (two doses), 3) AmBisome 10 mg/kg on day 1, AmBisome 5 mg/kg on days 3 and 7 (three doses), that demonstrates non-inferiority to the control arm in step 1 (in terms of EFA as defined above) will be considered for step 2. The ultimate decision will also take into account accrued safety, efficacy and mortality data and the results of PK/PD analysis. Whilst formal non-inferiority testing of EFA from step 1 will be the primary evidence used to inform the decision, the final choice of treatment arm will be at the discretion of the TSC.

Primary and secondary outcomes for step 1 and step 2 are as listed above. For step 2 the primary endpoint is mortality within the first 10 weeks by treatment arm. The two treatment groups will be compared using the Cochran-Mantel-Haenszel test for categorical data. Patients lost to follow up will be censored in the initial analysis. Sensitivity analysis in which all patients lost to follow up are assumed to have died, and Cox regression time-to-event analysis will be performed.

### Dissemination of results

The results from the different centres will be analysed together, published as soon as possible, and presented at an international conference. Step 1 and step 2 will be published in two separate reports. The Trial Management Group (TMG) will form the basis of the Writing Committee and will advise on the nature of the publication. The names of all investigators will be included in the authorship of any publication. Any authorship policy will be agreed by all investigators before the commencement of the trial. The members of the TSC and DMC will be listed with their affiliations in the acknowledgements or appendix section of the main publication. The funders will have no role in the decision to publish or the content of the publication.

### Ethical approval

The Research Ethics Committees of the London School of Hygiene and Tropical Medicine (6544–01), Botswana MoH HRDC (PPME-13/18/1 Vol IX (6)), PMH IRB (5/79/90a), and the University of Pennsylvania (820127) have approved the protocol. We will not begin any recruitment in any individual centre until local ethical approval has been obtained. Any further amendments will be submitted and approved by each ethics committee.

### Timeline

We anticipate that 60 patients will be enrolled in the study in Botswana and 160 patients in Tanzania over a 2-year period. This is feasible based upon historical numbers of admissions and previous studies at these sites [3, 4, 6].

### Ancillary studies

PK/PD and cost-effectiveness studies will be performed as outlined in this protocol. Cryptococcal isolates will be saved and shared with members of the cryptococcal research community for ongoing phenotypic and molecular epidemiology studies. Blood and CSF samples will be saved for ongoing studies examining the phenotype of the immune response to cryptococcus, and the pathophysiology of immune reconstitution inflammatory syndromes. Blood will also be stored for future studies determining how polymorphisms in immune response genes relate to susceptibility to and outcomes from cryptococcal meningitis. Any samples that require shipment will be in accordance with the local Material Transfer Agreement (MTA) guidelines.

### Quality control and assurance

The study sponsor is St George’s University of London. The sites will be visited at regular intervals in order to monitor the conduct of the trial and ensure that the principles of International Conference of Harmonisation (ICH) good clinical practice (GCP) is being adhered to. These visits will be made by the trial manager/monitor from the sponsoring institution. The frequency of monitoring visits will be according to need but will be at least after recruitment of the first 10 to 15 patients at each site, and after recruitment of 50 % and 100 % of patients (trial closure). If issues arise the frequency of visits will be increased. Site initiation assessments will be conducted at each site prior to trial initiation. The trial manager will visit the sites and ensure that all training (including GCP and protocol-specific training) has been completed, that drug supply and equipment are in place and that all study staff are up to date on the protocol and procedures, and are competent to undertake the roles described for them in the standard operating procedures (SOPs). An external audit by the London School of Hygiene and Tropical Medicine will also be conducted towards the beginning of the trial. Central monitoring will be performed in addition to the on-site monitoring procedures. Cumulative monthly reports (including information on recruitment rates, withdrawals, losses to follow up) will be compiled by the trial manager/statistician and reviewed by the TMG. Bimonthly reports on grade III/IV adverse events (AEs)/serious adverse events (SAEs)/serious adverse reactions (SARs)/suspected unexpected serious adverse reaction (SUSARs) will be compiled by the trial manager/statistician and reviewed by the TMG.

### Data collection and data management

Data will be managed using the Clinical DataFax System (Infectious Diseases Institute, Uganda). The study teams will directly enter data onto paper CRFs that will be labeled with unique patient identifiers. These will be scanned and sent in real time to the DataFax Research Unit, where data entry, verification and review will be performed. Routine quality control checks will be in place that will capture possible inconsistencies/missing data in real time, allowing queries to be sent back weekly to the study teams. Additionally, at monitoring visits the data entered in the CRFs will be checked against available source data by the trial monitor/manager. Trial data will be checked for missing or unusual values (range checks) and checked for consistency. If any such problems are identified, any data that are changed will be crossed through with a single line and initialled. Site investigators will not have access to cumulative data by study arm. On completion of recruitment, data will be securely exported from the DataFax database into the Stata statistical package, and following data cleaning and checking, the database will be locked and analysis will proceed according to the predefined analysis plan.

### Confidentiality

The principles of the UK Data Protection Act will be followed regardless of the countries where the trial is being conducted. The study site records will contain names, addresses, and contact details of the patients to enable follow up of patients. Unique numeric identifiers will be assigned to all patients, and only this unique number will be included in data sent from the trial sites and entered into the study database. The site investigator will ensure safekeeping of the patient data.

### Termination of the study

The trial will be considered closed when the last patient has completed 10 weeks in the study and all follow-up and laboratory reports have been received. Early termination could occur if the DMC decides there is an unacceptable level of adverse events in any of the test arms.

### Indemnity

The sponsor of the trial is St George’s University of London. All personnel involved in the trial will be expected to be indemnified by their employing authority. Patients will be indemnified through the policy of the trial sponsor. Local insurance will be taken out where local regulations require this.

## Discussion

The potential impact of a safe, sustainable regimen of high-dose intermittent AmBisome of equivalent efficacy to 2 weeks of daily Amphotericin-based induction (either conventional or liposomal), would be to reduce 10-week mortality in the majority of the African centres that still rely on fluconazole monotherapy, from approximately 60 % [5, 6, 14] to the 20−40 % seen with Amphotericin B-based combinations [2, 3]. It would also facilitate effective treatment, reduce the duration of hospital admissions, and lower the high burden of drug-related side effects currently seen in centres using conventional AmB-d based therapy. A large reduction in the number of doses, and duration of hospitalisation, together with reduced pricing of AmBisome, may result in costs that are comparable, or less than 2 weeks conventional AmB-d being given with appropriate monitoring.

## Trial status

The study is funded through a Gilead investigator initiated award to the Chief Investigators (Professor Harrison and Dr Jarvis). Funding for step 2 is to be confirmed. Trial recruitment is due to start in December 2014 pending the requisite ethical and regulatory clearance.

## Abbreviations

ALT: Alanine transaminases
AmB-d: Amphotericin B deoxycholate
AmBisome: Liposomal amphotericin B
ART: Anti-retroviral therapy
CFU: Colony-forming units
CRF: Case report form
CSF: Cerebrospinal fluid
DMC: Data Monitoring Committee
EFA: Early fungicidal activity
FBC: Full blood count
GCP: Good clinical practice
HRDC: Human Research and Development Committee
ICH: International Conference on Harmonisation
IRB: Institutional review board
iv: Intravenous
LP: Lumbar puncture
MoH: Ministry of Health
PD: Pharmacodynamics(s)
PK: Pharmacokinetic(s)
PMH: Princess Marina Hospital
SAE: Serious adverse event
TMG: Trial Management Group
TSC: Trial Steering Committee
